# Impact of PReOperative Midazolam on OuTcome of Elderly patients (I-PROMOTE): study protocol for a multicentre randomised controlled trial

**DOI:** 10.1186/s13063-019-3512-3

**Published:** 2019-07-15

**Authors:** Ana Kowark, Rolf Rossaint, András P. Keszei, Petra Bischoff, Michael Czaplik, Berthold Drexler, Peter Kienbaum, Moritz Kretzschmar, Christopher Rex, Thomas Saller, Gerhard Schneider, Martin Soehle, Mark Coburn, I-PROMOTE study group, Julia Van Waesberghe, Sebastian Ziemann, Anja Winkler, Maria Wittmann, Jonas Hinterberg, Maximilian S. Schaefer, Ulrich Frey, Jens Schreiber, Josef Briegel, Patrick Möhnle, Catharina Zeuzem, Markus Heim, Barbara Kapfer, Stefanie Pilge, Hansjörg Haas, Christopher Rex, Carmen M. Ring, Simone Traub

**Affiliations:** 1 Department of Anaesthesiology, Medical Faculty RWTH Aachen University, Aachen, Germany; 2 Center for Translational & Clinical Research Aachen (CTC-A), Medical Faculty RWTH Aachen University, Aachen, Germany; 3 Department of Anaesthesiology, Surgical Intensive Care, Pain and Palliative Care, Marien Hospital Herne, University Hospital of Ruhr University Bochum, Herne, Germany; 4 Department of Anaesthesiology and Intensive Care, University Hospital Tübingen, Tübingen, Germany; 5 Department of Anaesthesiology, University Hospital Düsseldorf, Düsseldorf, Germany; 6 Department of Anaesthesiology and Intensive Care Medicine, Otto-von-Guericke-University Magdeburg, Magdeburg, Germany; 7 Department for Anaesthesiology, Intensive Care, Emergency Medicine, Pain Therapy and Palliative Care, Kreiskliniken Reutlingen, Reutlingen, Germany; 8 Department of Anaesthesiology, University Hospital, LMU Munich, Munich, Germany; 9 Department of Anaesthesiology, Technical University of Munich (TUM), Munich, Germany; 10 Department of Anaesthesiology and Intensive Care Medicine, University Hospital Bonn, Bonn, Germany

**Keywords:** General anaesthesia, Midazolam, Patient satisfaction, Placebo, Premedication

## Abstract

**Introduction:**

Premedication of surgical patients with benzodiazepines has become questionable regarding risk-benefit ratio and lack of evidence. Though preoperative benzodiazepines might alleviate preoperative anxiety, a higher risk for adverse events is described, particularly for elderly patients (≥ 65 years). Several German hospitals already withhold benzodiazepine premedication from elderly patients, though evidence for this approach is lacking.

The patient-centred outcome known as global postoperative patient satisfaction is recognised as a substantial quality indicator of anaesthesia care incorporated by the American Society of Anesthesiologists. Therefore, we aim to assess whether the postoperative patient satisfaction after premedication with placebo compared to the preoperative administration of 3.75 mg midazolam in elderly patients differs.

**Methods:**

This study is a multicentre, randomised, placebo-controlled, double-blinded, two-arm parallel, interventional trial, conducted in nine German hospitals. In total 614 patients (≥ 65–80 years of age) undergoing elective surgery with general anaesthesia will be randomised to receive either 3.75 mg midazolam or placebo.

The primary outcome (global patient satisfaction) will be assessed with the validated EVAN-G questionnaire on the first postoperative day. Secondary outcomes will be assessed until the first postoperative day and then 30 days after surgery. They comprise among other things: functional and cognitive recovery, postoperative delirium, health-related quality of life assessment, and mortality or new onset of serious cardiac or pulmonary complications, acute stroke, or acute kidney injury.

Analysis will adhere to the intention-to-treat principle. The primary outcome will be analysed with the use of mixed linear models including treatment effect and study centre as factors and random effects for blocks. Exploratory adjusted and subgroup analyses of the primary and secondary outcomes with regard to gender effects, frailty, pre-operative anxiety level, patient demographics, and surgery experience will also be performed.

**Discussion:**

This is, to the best of our knowledge, the first study analysing patient satisfaction after premedication with midazolam in elderly patients. In conclusion, this study will provide high-quality data for the decision-making process regarding premedication in elderly surgical patients.

**Trial registration:**

ClinicalTrials.gov, NCT03052660. Registered on 14 February 2017. EudraCT 2016-004555-79.

**Electronic supplementary material:**

The online version of this article (10.1186/s13063-019-3512-3) contains supplementary material, which is available to authorized users.

## Background

Preoperative benzodiazepines are frequently applied to relieve patients’ preoperative anxiety and to enhance their satisfaction worldwide—not just in Germany. The causes of preoperative anxiety are multifactorial and have individually varying influences on the perioperative outcome [1]. Cognitive and behavioural changes, physiological reactions, different requirements for anaesthetic drugs and perception of pain, mood swings, wound-healing problems, and alteration of the immune system have been reported [2]. A generalised anxiety disorder was also significantly associated with major adverse cardiovascular and cerebrovascular events in patients undergoing coronary artery bypass graft surgery [3].

Postoperative patient satisfaction as a patient-centred outcome is recognised as a key quality indicator of anaesthesia care incorporated by the American Society of Anesthesiologists [4]. Anxiety is one of the multiple factors influencing patient satisfaction [5, 6], but it is not explicitly reported as a quality indicator. Provision of comprehensive preoperative information and patient involvement in the decision-making process are some important strategies for preoperative reduction of anxiety levels [6]. The effect of benzodiazepines on reducing preoperative anxiety remains controversial [7]. Therefore, the indiscriminate necessity of benzodiazepine premedication is questionable in regard to the risk-benefit assessment. Recent data in younger patients showed that there is an urge to reconsider the purpose of midazolam premedication [7]. Dose-dependent sedation leading to respiratory depression and decreased blood pressure are possible [7]. Further, paradox reactions and anterograde amnesia are unpleasant effects experienced by some patients [8, 9]. Also, incidence of pneumonia with an increased mortality was associated with the intake of benzodiazepines [10–12]. Postoperative delirium (POD) in elderly patients (> 65 years) is a serious complication with frequently lethal consequences. Overall, 13–50% of non-cardiac surgical patients experience POD [13]. The reasons are multifactorial [14], but 30–40% of the POD cases could be avoided by preventive measures. These include the avoidance of benzodiazepines, as they potentially enhance and prolong POD and cognitive dysfunction [13, 15]. In contrast, preoperative anxiety in elderly patients (> 65 years) is not associated with an increased risk for POD [16]. A non-pharmacological treatment of preoperative sleeping disorders and anxiety is recommended in these patients [13]. This is underlined in the American Geriatrics Society guideline for POD in elderly patients, which advises to avoid delirium-causing drugs including benzodiazepines [17].

A recently conducted randomised, placebo-controlled study in France included 1062 elective surgical patients < 70 years (mean 50 years) and showed no difference in regard to patient satisfaction among three groups (2.5 mg lorazepam, placebo, and no premedication) [18]. The time until extubation and early postoperative recovery were significantly prolonged and worse, respectively, in the lorazepam group than in the control or placebo group. Only 24% of the patients showed an increased preoperative anxiety level, and a subgroup analysis of these patients did not reveal a difference in regard to overall patient satisfaction. Of note, this study analysed premedication with lorazepam and excluded patients > 70 years.

Currently, in Germany, preoperative application of benzodiazepines in elderly patients is an important and controversial subject. On one hand, several hospitals in Germany already withhold benzodiazepine premedication from elderly patients, and on the other, some hospitals provide indiscriminate premedication with midazolam in all surgical patients < 80 years, notwithstanding the insufficient evidence for doing so [19]. Thus, a large randomised controlled trial (RCT) is indicated to assess the effect of premedication with midazolam on elderly patient satisfaction.

### Aims and objectives

We aim to analyse the self-reported experience of elderly patients after preoperative premedication. Our primary objective is to evaluate whether the global patient satisfaction on the first postoperative day is different in elderly patients with preoperative administration of placebo compared to midazolam (3.75 mg). Our secondary objectives are to evaluate whether other perioperative outcomes (e.g. POD, functional and cognitive recovery, health-related quality of life, and longer-term serious outcomes within 30 postoperative days) differ and depend on pre-existing patient characteristics (such as e.g. preoperative anxiety or frailty). The Impact of PReOperative Midazolam on OuTcome of Elderly patients (I-PROMOTE) study will be the first multicentre RCT analysing patient satisfaction in elderly surgical patients and will provide high-quality data for the decision-making process regarding premedication in these patients. We aim to generate clinically relevant decision support for premedication with benzodiazepines in elderly patients.

### Trial design

This is a protocol for a multicentre, double-blinded, randomised, two-arm parallel group, placebo-controlled, interventional clinical study. The randomisation is performed as a block randomisation, stratified by centre with 1:1 allocation.

We report our protocol in accordance with the Standard Protocol Items: Recommendations for Interventional Trials (SPIRIT) guidelines (Additional file 1) [20] and the Template for Intervention Description and Replication (TIDieR) to guide the reporting of components of our intervention (Additional file 2) [21].

## Methods

### Participants, interventions, and outcomes

#### Study setting

This multicentre RCT is conducted in nine German hospitals, which are listed on ClinicalTrials.gov NCT03052660. The site selection included university hospitals of tertiary care as well as hospitals of secondary care, in order to generate more generalisable results.

#### Study duration

The duration of subject participation is 31 days (from anaesthesia induction until the 30th postoperative day).

The study duration in total is expected to comprise about 24 months including evaluation and manuscript drafting. The recruitment period is expected to last 18 months, followed by a follow-up period of 1 month and 6 months for data cleaning, processing, analysis, and reporting. Patient recruitment started in October 2017. The study will be terminated after inclusion of the planned sample size of patients.

#### Eligibility criteria for study sites

The study sites were recruited within members of the Scientific Committee of Neuroanaesthesia of the German Society of Anaesthesiology and Intensive Care Medicine (DGAI).

#### Eligibility criteria for participants

Subjects who fulfil all of the following inclusion criteria are suitable for participation in the study:

1. Only legally competent patients
2. Written informed consent provided prior to study participation
3. 65–80 years, both genders
4. Elective surgery
5. Expected surgery duration ≥30 min
6. Planned general or combined regional and general anaesthesia
7. Planned extubation at the end of surgery (this criterion also comprises the removal of a laryngeal mask)

Subjects who fulfil one or more of the following exclusion criteria will not be included in the study:

1. Age > 80 years
2. Age < 65 years
3. Non-fluency in German language
4. Alcohol and/or drug abuse
5. Chronic benzodiazepine treatment
6. Intracranial surgery
7. Local and standby anaesthesia or solely regional anaesthesia
8. Monitored anaesthesia care
9. Cardiac surgery
10. Ambulatory surgery
11. Repeated surgery
12. Contraindications for benzodiazepine application (e.g. sleep apnoea syndrome, severe chronic obstructive pulmonary disease, allergy)
13. Allergy against any component of the placebo (lactose monohydrate, cellulose powder, magnesium stearate, microcrystalline cellulose) or investigational drug (midazolam, lactose) or the capsules (gelatine, E171 titanium dioxide, E132 indigotine)
14. Expected benzodiazepine requirement after surgery
15. Expected continuous mandatory ventilation after surgery
16. Patients who explicitly request anxiolytic premedication
17. Patients with severe neurological or psychiatric disorders
18. Refusal of study participation by the patient
19. Parallel participation in interventional clinical studies within the previous 30 days

#### Recruitment

Patients will be recruited consecutively during the preoperative anaesthesia consultation in the clinical routine by an investigator, with the support of the attending anaesthetists. Each participating centre will recruit as many patients as possible. The time point of informed consent will be documented, to enable verification of the patient recruitment and randomisation sequence, in order to prevent selection bias. All screened patients (including the screening failures and enrolled patients) will be documented in a screening/enrolment log.

Strategies to enhance recruitment rates will include newsletters and telephone calls on a regular basis. Furthermore, the publication policy will further motivate the participating centres, as the authorship will depend on the number of enrolled and completely documented patients.

#### Allocation

**Fig. 1:**
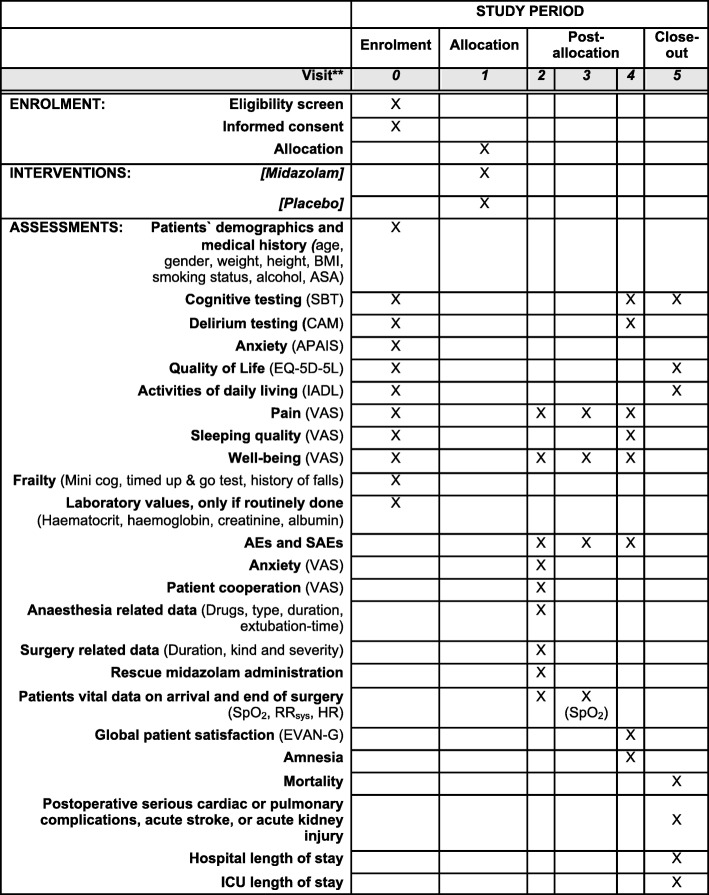
Participant timeline according to the SPIRIT Statement. **Visit 0: preoperative screening and baseline visit, Visit 1: 30–45 min before surgery, Visit 2: operating room, Visit 3: surgery day postoperatively within 0.5–1.5 h after surgery, Visit 4: first postoperative day; Visit 5: 30th postoperative day. *AE* adverse event, *APAIS* Amsterdam Preoperative Anxiety and Information Scale, *ASA* American Society of Anesthesiologists physical status, *BMI* body mass index, *CAM* Confusion Assessment Method, *EVAN-G* Evaluation du Vécu de l’Anesthésie Générale, *EQ-5D-5 L* health-related quality of life assessment, *IADL* Instrumental Activities of Daily Living scale, *ICU* intensive care unit, *RRsys* systolic blood pressure, *SAE* serious adverse event, *SBT* Short Blessed Test, *SpO2* peripheral oxygen saturation, *VAS* visual analogue scale

Sequence generation for randomisation will be carried out using a computer-based approach [22] by the biostatistician (APK) of the Department of Medical Informatics RWTH Aachen University Hospital. A randomisation stratified by study centre will be implemented. Sequences will be generated using a 1:1 ratio of the treatment arms and a permuted block randomisation. To ensure allocation concealment, the block sizes and allocation sequence will be concealed from all investigators and staff throughout the study until after the database lock. The allocation sequence list will be provided only to the pharmacy directly by the biostatistician. The Department of Pharmacy, University Medical Center Johannes Gutenberg University Mainz, Germany will provide sealed, opaque containers holding the assigned treatment to each centre. These containers will be labelled with the ascending unique randomisation numbers. After the recruitment and enrolment of a patient by an investigator, the investigator is obliged to take the next consecutive medication container with the ascending randomisation number at visit 1; see the following discussion and Fig. 1. The investigator will assign this unique randomisation number together with the respective medication container to this enrolled patient. In practice this means that the medication container will be handed out to an independent nurse who is responsible for this next patient (see the description of the intervention in the following section).

For emergency un-blinding, all centres will receive opaque, sealed emergency envelopes including the information about the assigned treatment by the pharmacy.

#### Intervention

Patients who have met all inclusion criteria and none of the exclusion criteria will be randomly assigned to receive an oral premedication with either 3.75 mg midazolam or placebo. Premedication will be administered once, 30–45 min before the estimated surgery time point, as recommended in the summary of product characteristics for midazolam and usually performed at the participating sites. The investigational products are encapsulated and packed into single small, opaque, sealed and relabelled containers by the Department of Pharmacy, University Medical Center Johannes Gutenberg University Mainz, Germany according to the MHRA (Medicines and Healthcare Products Regulatory Agency). Study investigators will have to take the next consecutive container and note the patient identification number on a prescribed space on its label. Thereafter, the investigator will hand out the respective container to an independent nurse who is responsible for the patient but not involved in the study. The principal investigator (PI) will inform the entire ward staff of the different units in the hospital about the performance of this study before its initiation. The responsible nurse will also be informed each time a patient is enrolled. The nurse will be advised to hand out the respective container to the patient face to face, as is usually done in the clinical routine. The only difference is that the container contains a capsule and in the clinical routine the patients would receive a tablet. Specific training for this procedure is not necessary. The patients have to take the medication with a small sip of water. The location of the intervention intake will be either the standard care ward of the patient or the patient preparation room, depending on the standard operating procedure (SOP) of the respective participating site.

#### Intervention: adherence

Intervention adherence will be assessed by storage of the empty container for each patient by the respective nurse. The monitoring team will check the entered patient identification number and the randomisation number on the container and crosscheck it with the enrolment sequence.

#### Intervention: modifications

In accordance with the requirement of our ethics committee, patients with apparent or verbally expressed anxiety might receive additional midazolam intravenously (i.v.) when entering the surgery area, according to the clinical routine (study- and group-independent). This midazolam will be applied carefully titrated (at 0.5 mg) i.v., by the attending anaesthetist under monitoring of the patients’ vital data, according to the SOP of the respective department. Additional i.v. administered “Rescue” midazolam will be noted in the patient’s file. These patients will be retained in the study and followed up to prevent missing data, according to the intention-to treat (ITT) principle. Of note, the preoperative anxiety level, which is measured at operating room admission, will be recorded before administration of this “Rescue” midazolam.

#### Intervention: concomitant care

After patient inclusion, the entire ward staff will be informed and it will be noted in the patient files that the patient should not receive any benzodiazepine in the clinical routine, if not indispensable until the surgery. Other medications may be provided as usual in routine care. Anaesthetic and surgical management will be performed according to the clinical routine, without any study-specific restrictions.

#### Outcomes

##### Primary outcome measure

Global patient satisfaction will be evaluated with the self-report EVAN-G (Evaluation du Vécu de l’Anesthésie Générale) questionnaire [23] on the first postoperative day, at visit 4 (see Fig. 1). The EVAN-G is a validated questionnaire comprising 26 items within six dimensions (attention, information, privacy, pain, discomfort, and information) which is used to assess perioperative patient satisfaction within the first 48 h after surgery.

##### Secondary outcome measures

The secondary outcome measures are as follows:

- Assessment of preoperative frailty within our patient population and adjusted subgroup analysis of the primary outcome depending on the patient’s frailty. Frailty assessment will be performed according to Oresanya et al. [24]. This includes, in addition to the assessment of the medical history and laboratory values, the history of falls, the Mini-Cog test [25], and the timed “Up & Go” test [26].
- Analysis of the relationship of preoperative frailty and the other assessed postoperative outcomes
- Assessment of the impact of premedication on the patients’ functional and cognitive recovery (difference in proportion of patients). Functional ability will be assessed by the Instrumental Activities of Daily Living (IADL) scale [27] (recovery is defined as change between baseline and day 30 after surgery). Cognitive status will be assessed by the Short Blessed Test (SBT) [28] (recovery is defined as change between baseline and day 1 and day 30 after surgery). The SBT was chosen for the cognitive assessment, as it can also be applied by phone on postoperative day 30
- Assessment of the impact of premedication on POD (difference in proportion of patients). Delirium will be assessed by the Confusion Assessment Method (CAM) [29] or the CAM-ICU for patients in the intensive care unit [30]. Delirium will be assessed at baseline and on the first postoperative day.
- Assessment of the impact of premedication on the perioperative condition of well-being, pain, and sleeping. These outcomes will be assessed with the visual analogue scale (VAS, values 0–100, with 100 corresponding to best well-being, worst pain, and best sleeping). These data will be assessed at baseline, in the operating room, 0.5–1.5 h after surgery, and on the first postoperative day.
- Assessment of the impact of premedication on patient cooperation directly preoperatively. Patient cooperation will be rated by the attending anaesthetist (via VAS, with 100 corresponding to the best cooperation)
- Assessment of the impact of premedication on the patient’s anxiety at arrival in the operating room (rated via VAS by the patient, with 100 corresponding to the strongest anxiety). A cut-off value of 72 mm will indicate high anxiety [31].
- Assessment of the difference in the proportion of patients with rescue midazolam application before surgery
- Assessment of the difference in the proportion of patients with adverse vital data values upon arrival into the operating room, after extubation and 0.5–1.5 h later
- Assessment of the difference in time to extubation depending on the premedication. The attending anaesthetist will measure this time from cessation of the anaesthesia until extubation
- Assessment of the difference between the groups regarding the change of the health-related quality of life from baseline until postoperative day 30. This outcome will be measured with the EQ-5D-5 L [32].
- Difference between the two groups in the proportion of the longer-term outcomes mortality or the new onset of serious cardiac or pulmonary complications, acute stroke, or acute kidney injury within 30 postoperative days. Outcomes will be defined according to the following definitions:
  1. ***Serious cardiac complications.*** *Cardiac arrest*: The absence of cardiac rhythm or presence of pulseless electrical activity requiring the initiation of cardioplulmonary resuscitation, which includes chest compression. *Myocardial infarction*: Electrocardiography changes, new elevation in troponin, or physician diagnosis. Signs of myocardial infarction in the autopsy
  2. ***Serious pulmonary complications.*** *Pneumonia*: Clinical or radiological diagnosis. *Pulmonary embolism*: Radiological diagnosis. Signs of pneumonia or pulmonary embolism in the autopsy
  3. ***Acute Stroke.*** Defined as a new focal or generalised neurological deficit of > 24 h duration in motor, sensory, or coordination functions with compatible brain imaging and confirmed by a neurologist. Transient ischemic attack is not considered as acute stroke. Signs of stroke in the autopsy
  4. ***Acute kidney injury.*** Defined according to the Acute Kidney Injury Network (AKIN) classification [33] as AKI stage ≥ 2. This means a greater than two to three times increase of creatinine from baseline within the hospital stay, urine output less than 0.5 ml kg^− 1^ per hour for more than 12 h, or signs of acute kidney injury in the autopsy.

After hospital discharge, events will only be defined as present if they led to hospital re-admission or death.

- Adjusted subgroup analysis of the primary outcome depending on the preoperative baseline anxiety level, the patient demographics, surgery experience of the patients, and gender effects. Baseline anxiety will be assessed preoperatively by the self-reported German version of the Amsterdam Preoperative Anxiety and Information Scale (APAIS) [34]. Patients with a cut-off value of 12 will be considered as anxious, as proposed by Berth et al. [34]
- Difference between the two groups in the proportion of adverse events (AEs) and serious adverse events (SAEs) according to the medical charts until postoperative day 30
- Assessment of the proportion of patients with amnesia on the first postoperative day
- Assessment of the impact of premedication on the hospital length of stay (LOS) and intensive care unit (ICU)-LOS. Difference of the durations between the two study groups.

#### Participant timeline

The time schedule of enrolment, interventions, assessments, and visits for participants is presented in Fig. 1.

##### Visit 0 (baseline visit)

After receiving study-specific patient information and written informed consent, the investigator will perform a baseline visit, which includes the assessment of the patient demographics, medical history, and the most recent preoperative routine laboratory values (only if done in the clinical routine). The study-specific baseline testing (anxiety, cognitive, and functional assessment, health-related quality of life assessment, pain, sleeping, and well-being) and frailty assessment will also be performed. The patient will receive the next consecutive randomisation number.

##### Visit 1 (surgery day, preoperative)

At 30–45 min before surgery, eligible and enrolled patients will receive the assigned container including the allocated treatment (relabelled concealed capsule including midazolam or placebo).

##### Visit 2 (surgery day, intraoperative)

Patient cooperation and anxiety will be evaluated at patient admission into the operating room via the VAS. Anaesthesia will be conducted according to the clinical routine, including the kind of anaesthesia and the airway device used. Intraoperative surgery- and anaesthesia-related data will be assessed. An additional application of benzodiazepines is not desired, but left to the discretion of the attending anaesthetist, who will be blinded to the allocation treatment. The attending anaesthetist will measure the time until extubation or removal of the airway device after cessation of the anaesthetic agent (inhalative or intravenous), respectively. The patient will be questioned about pain and well-being after surgery at operating room departure via the VAS.

##### Visit 3 (surgery day, postoperative)

The patient will undergo further study-specific assessments in the post-anaesthesia care unit or ICU. Postoperative analgesia will also be assessed until visit 3.

##### Visit 4 (first postoperative day)

A follow-up visit with study-specific assessments will be performed on the ward or ICU.

##### Visit 5 (30th postoperative day)

A follow-up visit with study-specific assessments will be performed via telephone or visit on ward, if the patient is still in hospital. The hospital LOS and ICU-LOS data will be collected from the hospital database.

### Sample size

The sample size was calculated based on detecting a minimum 5-unit difference in the primary outcome variable overall patient satisfaction measured with the EVAN-G. Assumptions regarding the standard deviation of EVAN-G in the population were based on previous work [23]. Setting a type 1 error of 0.05 and a power of 0.8 and assuming the standard deviation of EVAN-G to be 14 units, 248 patients per group are needed to detect a 5-unit difference.

Considering a drop-out rate of 10% and a screening failure of 10%, we decided to include 614 patients in total (3.75 mg midazolam *n* = 307 and placebo *n* = 307).

### Blinding

This study is planned in a double-blinded manner. The investigator, the intraoperative attending anaesthetist, and the patient will not be aware of the treatment allocation in all cases, as the medication will be encapsulated and provided by an independent nurse.

### Un-blinding procedures

In the event of a medical emergency, which requires identification of an individual patient’s treatment, the investigators are permitted to open the respective emergency envelope. A justification must be documented in the patient’s medical record and in the case report form (CRF). Un-blinding is not necessary in case of additional preoperative midazolam treatment under controlled conditions in the clinical routine (see “Intervention:modifications”).

### Data collection methods/data management

First, all collected patient data during this clinical study will be entered and/or filed in the respective patient CRF. The patient’s study participation must be documented appropriately in the patient CRF with study number, subject number, date of subject information and informed consent, and date of each visit. Source data should be filed according to the Good Clinical Practice (GCP) guidelines. The sponsor’s data manager will be responsible for data processing, according to the sponsor’s SOPs. Database lock will occur only after quality assurance procedures have been completed.

Second, the investigators will transcribe all information required by the protocol into a web-based electronic data collection system OpenClinica [35] electronic case report form (eCRF). The eCRF will be developed by the data manager for the study. Detailed information on the eCRF completion will be provided during the site initiation visits via an eCRF completion manual and an e-learning tool. The access to the e-learning tool and to the eCRF will be password controlled. Plausibility checks will be performed according to a data validation plan. Inconsistencies in the data will be queried to the investigators via the electronic data collection system; answers to queries or changes of the data will be documented directly into the system. Plausibility checks will be performed to ensure correctness and completeness of these data. By signing the CRF (eCRF/eSignature), the investigator confirms that all investigations have been completed and conducted in compliance with the clinical study protocol, and that reliable and complete data have been entered into the eCRF.

### Quality control

Standardisation procedures will be implemented to ensure accurate, consistent, complete, and reliable data, including methods to ensure standardisation among sites (e.g. training, newsletters, investigator meetings, monitoring, centralised evaluations, and validation methods). To prepare the investigators and to standardise performance, training will be held during the study initiation visit for each centre before study start. Manuals for standardised conduction of interviews will be provided to the investigators.

The PI of each centre will ensure adequate qualification and information about the study of all sub-investigators and the assisting study personnel. The PI will maintain a study staff authorisation log, with listed responsibilities of each person.

### Record keeping

Essential documents which comprise, among others, study subject files, the subject identification code list, and signed informed consent forms, should be archived for at least 10 years. The PI should take measures to prevent accidental or premature destruction of these documents.

### Retention

After inclusion and randomisation of the patient, the study site will make every reasonable effort to follow the patient for the entire study period. We do not expect a high loss to follow-up or missing data for most outcomes (including the primary outcome), as most assessments are finished on the first postoperative day. To enhance participant retention for the 30 days follow-up, the investigators will schedule an appointment for the telephone call and verify the correctness of the phone number before patient discharge from hospital. Appointment reminders will be set in electronic calendars.

Patients may withdraw at any time from this study in whole or in part. Investigators must ask the patient if he/she is willing to continue participation for further follow-up assessments.

### Statistical methods: outcomes

Primary analysis of the study outcome will be performed according to the ITT principle. The ITT analysis will also include the patients who have received additional “Rescue” i.v. midazolam preoperatively on behalf of the attending anaesthetist during the clinical routine. The exact pre-specification of the full analysis set will be performed based on a blinded data review. According to the International Council for Harmonisation of Technical Requirements for Pharmaceuticals for Human Use (ICH)-E9 guideline, patients who received no treatment can be excluded if the decision to treat or not to treat is not influenced by the knowledge of the assigned treatment. All reasonable efforts will be made to evaluate the primary endpoint in all study subjects regardless of adherence to the study protocol. If it is not possible to perform the EVAN-G test on the first postoperative day, the test must be performed on the next possible day. A per protocol (PP) data set will be defined for secondary analyses, composed of all randomised patients who have no major protocol deviations throughout their whole study period. Safety variables will be analysed on a data set comprising all study subjects who have received study medication. Descriptive analyses of all study data will be performed for both treatment arms. Frequencies for categorical variables and means, standard deviations and selected quantiles for quantitative variables, as well as frequencies of missing data will be tabulated. Distributions of variables will be graphically examined using appropriate visualisation tools.

The primary, confirmatory analysis will be performed on the EVAN-G global index measure using a linear mixed-effects model including treatment effect, study centre, and blocks, but no interaction terms. The treatment effect will be tested against a null hypothesis of no effect using an *F* test, and 95% confidence intervals for the treatment effect estimate will be calculated. Secondary analyses will be performed to explore gender-specific treatment effects, and the robustness of the results of the primary analysis will be explored by repeating the analysis on the PP data set and by imputation of missing primary endpoint data based on baseline characteristics.

These analyses of secondary outcomes will be considered exploratory and will be performed independently for each secondary outcome without adjustment for the multiple analyses. The outcomes functional ability, cognitive recovery, POD, use of rescue midazolam, adverse vital data, and presence of long-term outcomes, AE, and amnesia will be analysed as dichotomous outcome variables, and the difference in proportions between the treatment groups along with their standard errors will be calculated. The outcomes well-being, pain, and sleeping, which are measured using the VAS, will be analysed using linear mixed-effect models including treatment effect and treatment-time interactions. The outcomes patient cooperation, anxiety in the operation room, length of hospital and ICU stay, and time to extubation will be analysed as continuous outcome variables, and the means in each intervention group and differences in means will be calculated. Randomisation and data analysis will be carried out using the R language for statistical computing [22]. A detailed trial statistical analysis plan will be finalised before database lock.

### Statistical methods: additional analyses

Exploratory adjusted and subgroup analyses of the primary and selected secondary outcomes with regard to gender effects, frailty status, preoperative anxiety level, patient demographics, and surgery experience will also be performed. These analyses will be performed independently for each outcome without adjustment for multiple analyses. The explanatory factors will be analysed as dichotomous variables.

### Data monitoring

A formal Data Monitoring Committee will not be established for this study, which is performed during the clinical routine and implies minimal risks associated with the application of placebo instead of 3.75 mg midazolam.

This study will be monitored regularly by a qualified monitor from the Center for Translational & Clinical Research Aachen (CTC-A) —belonging to the sponsor— according to GCP guidelines and the respective SOPs. Monitoring procedures include study initiation visits and interim monitoring visits on a regular basis according to a mutually agreed schedule.

During these visits, the monitor will check for completion of the entries on the eCRF/CRF; for compliance with the clinical study protocol, GCP principles, and regulatory authority requirements; for the integrity of the source data with the eCRF/CRF entries; and for subject eligibility. Monitoring will also aim to detect any misconduct or fraud. In addition, the monitor will check whether all AEs and SAEs have been reported appropriately within the required time periods. Further details of monitoring activities will be described in the CTC-A’s monitoring manual.

### Interim analysis and stopping guideline

Interim analyses are not planned in this study.

The coordinating PI may decide together with the sponsor’s representative (CTC-A) to terminate this study entirely in case of a changed risk-benefit ratio, which indicates a premature study termination in order to protect the subject’s health.

The study will be prematurely terminated for an individual subject in case:

- The patient requests to leave or withdraws informed consent
- The patient did not meet the inclusion and/or exclusion criteria
- There is a patient condition which is incompatible with a premedication or any study procedure.

### Harms

Safety assessments will consist of monitoring and recording all AEs and SAEs and the regular monitoring of intraoperative vital data by the attending anaesthetist. All AEs will be defined according to the ICH-GCP guidelines; see Additional file 3.

Midazolam incorporates several side effects, which probably jeopardise the patient. Additional harms, other than the usually occurring side effects in the clinical routine are not expected in the midazolam group in this study. All possible side effects are described in the summary of (medicinal) product characteristics for midazolam. For the placebo group, we do not expect any significant harm, as in the case of strong preoperative anxiety or agitation, additional midazolam application may occur on behalf of the attending anaesthetist at any time.

### Auditing

Audits by the sponsor are not planned for this study, but a member of the sponsor’s quality assurance function may arrange a visit in order to audit the performance of the study at a study site. Auditors conduct their work independently of the clinical study and its performance. Inspections by regulatory authority representatives and institutional ethics committees (IECs) are possible at any time, even after the end of study. The investigator must inform the sponsor immediately about any inspection. The investigator and institution will permit study-related monitoring, audits, and reviews by the IEC and/or regulatory authorities, and will allow direct access to source data and source documents for such monitoring, audits, and reviews.

### Confidentiality

All subjects will be identified by a unique 7-digit patient identification number (xxx-yyyy) and randomisation number (xxx-RAND-yyyy). The first 3 digits indicate the centre, and the last 4 digits the ascending patient/randomisation number. Each PI will keep a list in a safe location which will allow the identification of the pseudonymised patients.

The patients’ informed consent forms, with their printed names and signatures, will be filed separately in the investigator’s site file (ISF). All source data and the ISF will be protected against unauthorised access in locked cabinets with restricted access under the responsibility of the PI of each participating centre.

Patients will be informed about data protection and the fact that data passed to other investigators or an authorised party for analysis will occur in a pseudonymised manner. Data analysis by the biostatistician will also be performed in a pseudonymised manner.

### Access to data

Access to encoded data or source documents will only be given to authorised bodies or persons (sponsor, authorised staff, auditors, competent authorities, or ethics committee members) for validation of data. Also in case of publication, confidentiality of collected data will be warranted.

Access to the online database will be restricted by personal passwords and may be checked via an audit trail which is implemented in the OpenClinica database system.

### Post-trial care

No specific post-study arrangements are made, and no specific post-study care will be performed after this study. All subjects will return to their standard routine medical care after the study as needed. This also applies to subjects who withdraw their consent during the course of the study.

### Dissemination policy

The study results will be published in appropriate international scientific journals and presented at scientific conferences, regardless of the results. A professional writing service will not be engaged. Details of the publication policy will be given in the clinical study agreement. The coordinating PI will additionally disclose study results in the ClinicalTrials.gov registry.

### Patient and public involvement

Patients or the public were not involved in the design of this study. Published results will be disseminated to the study participants on request.

## Discussion

Midazolam is a routinely used premedication in surgical patients worldwide, not just in Germany [7]. It is mostly applied to alleviate preoperative anxiety [36], but some anaesthesiologists might also use benzodiazepine premedication for prevention of intraoperative awareness, induction of sedation, haemodynamic stabilisation, and analgesia [7]. However, there is no medical evidence that benzodiazepine premedication is advantageous for all patients, especially the elderly ones. However, there is also no high-quality evidence indicating that withholding of preoperative midazolam in all elderly patients is beneficial. A Cochrane analysis of the anxiolytic premedication effect on time to discharge in a day case surgery setting found similar discharge times between patients with premedication compared to the placebo group, though impaired psychomotor function after benzodiazepines application was described [37]. Of note, this Cochrane analysis failed to report outcomes of efficacy of anxiolytic premedication, and the included studies were of poor quality and very heterogeneous. Therefore, a balanced judgement on the risks and benefits of premedication was hindered. Another Cochrane review showed that there is a lack of evidence for premedication effects in elderly patients [14]. I-PROMOTE aims to gain first evidence for this vulnerable patient group regarding the premedication effect on patient satisfaction and other outcomes. We think that the results of this study will be useful for justifying the waiving of indiscriminate premedication with benzodiazepines in elderly patients.

The decision to administer only 3.75 mg midazolam in this study instead of 7.5 mg is justified by the recommendation in the German summary of product characteristics for midazolam in elderly patients: “Elderly patients showed a larger sedative effect, therefore they may be at increased risk of cardio-respiratory depression as well. Thus, midazolam should be used very carefully in elderly patients, and if needed, a lower dose should be considered.” Administration of a reduced midazolam dose of 3.75 mg reflects the standard routine approach for elderly patients in Germany [38]. Exclusion of patients older than 80 years was based on the clinical routine of the participating centres, which generally do not administer midazolam in patients older than 80 years.

Our decision to choose the global postoperative patient satisfaction as the primary outcome is based on the increased importance for assessment of the patient-reported experience of healthcare as an important outcome [4, 6]. We acknowledge that patient satisfaction is influenced by several factors, e.g. preoperative anxiety, amnesia, pain, or surgical complications. Thus, we expect that the randomised design will enable an equal distribution of the aforementioned factors. Furthermore, we are going to record the postoperative analgesia requirement, pain, and amnesia as well as any AEs in this study.

One strength of this study is the double-blinded design. It will provide low-biased results and support high-quality study results. In contrast to the previous similar study in younger patients [18], a third parallel arm without any treatment was avoided, as blinding is an important part of the study design, and an arm without treatment cannot be blinded at the patient level.

One limitation is that we are not going to control the general anaesthesia regime (type, quantity, application time), but we think that this will provide more generalisable results. A further limitation is that the study results are not generalisable to institutions which use other kinds of benzodiazepines than midazolam or other drugs like α2-adrenoceptor agonists for premedication.

In conclusion, I-PROMOTE will provide high-quality data for the decision-making process regarding premedication with 3.75 mg midazolam in elderly patients.

### Trial status

This study has been recruiting since 12 October 2017, and it is estimated that the recruitment will end in May 2019. The protocol is version 2.0, dated 20 June 2017.

## Additional files

Additional file 1: SPIRIT 2013 checklist: recommended items to address in a clinical trial protocol and related documents. (DOCX 31 kb)

Additional file 2: The TIDieR (Template for Intervention Description and Replication) checklist. (PDF 121 kb)

Additional file 3: Definition of adverse events (AEs). (PDF 39 kb)

## Data Availability

Only the coordinating centre (University Hospital RWTH Aachen) will have access to the full final trial data set. The PIs from each participating centre will have access to their own site’s data sets. It is established in the study site agreements that an individual study site should not disclose the individual data sets prior to the main publication. The data sets used and/or analysed during the current study are available for the public from the corresponding author only on reasonable request.

## Abbreviations

AE: Adverse event
APAIS: Amsterdam Preoperative Anxiety and Information Scale
BfArM: Federal Institute for Drugs and Medical Devices, Germany
CAM: Confusion Assessment Method
CRF: Case report form
CTC-A: Center for Translational & Clinical Research Aachen
DGAI: German Society of Anaesthesiology and Intensive Care Medicine
eCRF: Electronic case report form
GCP: Good Clinical Practice
i.v.: Intravenously
IADL: Instrumental Activities of Daily Living
ICU: Intensive care unit
IEC: Institutional ethics committee
ISF: Investigator’s site file
ITT: Intention-to treat
LOS: Length of stay
MHRA: Medicines and Healthcare Products Regulatory Agency
PI: Principal investigator
POD: Postoperative delirium
PP: Per protocol
RCT: Randomised controlled trial
SAE: Serious adverse event
SBT: Short Blessed Test
SOP: Standard operating procedure
SPIRIT: Standard Protocol Items: Recommendations for Interventional Trials
TIDieR: Template for Intervention Description and Replication
VAS: Visual analogue scale
