# Supplementary material for: Impact of PReOperative Midazolam on OuTcome of Elderly patients (I-PROMOTE): study protocol for a multicentre randomised controlled trial
Source: Trials. 2019 Jul 15;20:430. doi: 10.1186/s13063-019-3512-3 (PMC6632125; doi:10.1186/s13063-019-3512-3)
Supplement: Supplementary file 3 — Definition of adverse events (AEs). (PDF 39 kb) [file 13063_2019_3512_MOESM3_ESM.pdf]

Additional File 4

Definition of adverse events (AEs)

All AEs will be defined according to the ICH-GCP guidelines as “any untoward medical occurrence, including an exacerbation of a pre-existing condition, in a patient or clinical investigation subject administered a pharmaceutical product and that does not necessarily have a causal relationship with this treatment”(ICH E6:1.2). All AEs have to be followed up until resolving or stabilisation at an acceptable level by the investigator.

Each AE will be reported on an Adverse Event Case Report Form and specified in regard to:

1. Its duration (start and end dates)

2. Its severity grade (mild, moderate, severe). The severity grade of an adverse event provides consists of a qualitative assessment of the extent or intensity of an adverse event, as determined by the investigator or as reported by the subject.

3. Its relationship to the study drug (suspected / not suspected). Medical judgment should be used to determine the relationship, considering all relevant factors, including pattern of reaction, temporal relationship, de-challenge or re-challenge, confounding factors such as concomitant medication, concomitant diseases and relevant history.

4. Treatment required and action taken with trial drug

5. Outcome

6. Seriousness

Definition of Serious Adverse Events (SAEs)

A SAE is defined as an adverse event that:

- Results in death (fatal);
- Is immediately life-threatening;
- Results in persistent or significant disability/incapacity;
- Requires or prolongs patient hospitalisation;
- Is a congenital anomaly/birth defect;

  or

- Based upon appropriate medical judgment, may jeopardise the patient and may require medical or surgical intervention to prevent one of the previously listed outcomes.

An event does not need to be reported as a SAE if it represents only a relapse or an expected change or progression of the condition that was the cause of treatment without any other symptoms and signs than those present before treatment. This type of event needs only to be reported as an AE.

Reporting procedures

AE and SAE reporting forms will be provided to each principle site investigator by the sponsor (CTC-A). All investigators will be trained on the AE/ SAE definition, documentation and reporting.

The principle investigator of each centre will have to report all SAEs to the sponsor (CTC-A) within 24 hours of discovery or notification of the event. The sponsor will collect all SAE reports and write the annual safety report.
